# Randomised controlled trial of school-based humanistic counselling for emotional distress in young people: Feasibility study and preliminary indications of efficacy

**DOI:** 10.1186/1753-2000-4-12

**Published:** 2010-04-22

**Authors:** Mick Cooper, Nancy Rowland, Katherine McArthur, Susan Pattison, Karen Cromarty, Kaye Richards

**Affiliations:** 1 University of Strathclyde, Glasgow, UK; 2 British Association for Counselling and Psychotherapy (BACP), Lutterworth, UK; 3 Newcastle University, Newcastle upon Tyne, UK

## Abstract

**Aims:**

The purpose of this study was to test the feasibility of a randomised controlled trial comparing six weeks of humanistic school-based counselling versus waiting list in the reduction of emotional distress in young people, and to obtain initial indications of efficacy.

**Methods:**

Following a screening procedure, young people (13 - 15 years old) who experienced emotional distress were randomised to either humanistic counselling or waiting list in this multi-site study. Outcomes were assessed using a range of self-report mental health measures, with the emotional symptoms subscale of the Strengths and Difficulties Questionnaire (SDQ) acting as the primary outcome indicator.

**Results:**

Recruitment procedures were successful, with 32 young people consenting to participate in the trial and 27 completing endpoint measures. Trial procedures were acceptable to all involved in the research. No significant differences were found between the counselling and waiting list groups in reductions in levels of emotional symptoms (Hedges' *g *= 0.03), but clients allocated to counselling showed significantly greater improvement in prosocial behaviour (*g *= 0.89) with an average effect size (*g*) across the nine outcome measures of 0.25. Participants with higher levels of depressive symptoms showed significantly greater change.

**Conclusion:**

This study suggested that a randomised controlled trial of counselling in schools is acceptable and feasible, although initial indications of efficacy are mixed.

**Trial registration:**

Current Controlled Trials ISRCTN68290510.

## Background

Levels of mental health problems in children and young people are increasing [1], with one in ten children in Britain now experiencing a diagnosable mental health disorder [2].

Within the UK, one of the responses to this growing problem has been the establishment of school-based counselling services [3]. Recent years have seen the establishment of universal post-primary school counselling provision in Northern Ireland and in secondary schools in Wales; and a policy commitment to providing access to school counselling to all pupils in Scotland by 2015 [4]. In contrast to school counseling and guidance in the US and Asia [5], UK provision tends to be based around a humanistic, person-centred model of practice [6-8], with a focus on young people's emotional difficulties (rather than educational attainment), and a predominance of one-to-one meetings with a counsellor rather than group therapy. Supporting such initiatives, a recent report by the Institute for Public Policy Research [6] concluded that, across the UK, there should be a counsellor in every school.

In terms of efficacy, the effect sizes observed in studies evaluating psychotherapeutic interventions versus no intervention with children and adolescents are around 0.70 [7,8]. In a study specifically evaluating school-based psychotherapy and counselling interventions [9] the effect size observed was 0.95. However, such evidence of efficacy primarily comes from trials of cognitive-behavioural therapies (CBT), and generally within a group format. Meta-analyses of person-centred approaches in child and adolescent psychotherapy have found effect sizes ranging from 0.15 to 0.93 [10].

With respect to emotional and affective problems, Birmaher et al. [11] found that 12-16 weeks of nondirective supportive treatment (similar to person-centred therapy) was associated with an 85% rate of remission from depression at two year follow-up - similar to CBT and systemic behaviour family therapy - although CBT was superior at 12-16 weeks [12]. However, in the Brent et al. study, it is not clear what role allegiance effects [13] played in reducing the apparent efficacy of person-centred therapy. The fact that around 1 in 5 young people did not remit from depression as a result of CBT [12] also indicates the need to develop and evaluate alternative interventions.

In terms of uncontrolled evidence regarding counselling in schools, a recent meta-analysis of data from 15 UK-based evaluation studies of person-centred or humanistic counselling in schools found a mean weighted effect size of 0.81 from pre- to post-counselling; with 82% of young people, on average, describing their counselling as 'helpful' or 'very helpful' [14]. Research from the UK also indicates that school-based counselling services are feasible to implement and are highly acceptable to young people, pastoral care coordinators and teachers [14-19]. While referrals to Children and Adolescent Mental Health Services (CAMHS) are currently available for young people within secondary schools who meet the necessary assessment and diagnostic criteria, school-based counselling provisions are perceived by children, parents, pastoral care staff and related professionals as an important additional resource: highly accessible; non-stigmatising; capable of responding quickly to young people's mental health needs; and of particular value to emotionally distressed and/or 'troubled' young people who may not be appropriate for referral to educational or clinical psychology services [14,16].

Whilst in the UK, then, there is a rapid growth of humanistic counselling in secondary schools, the strongest evidence to support it is currently correlational and based on clients' and teachers' perceptions. This suggests that a fully-powered randomised controlled trial of such an intervention is required. However, as such a study has yet to be carried out within a UK-context, it was considered essential to first conduct a pilot trial, to assess feasibility of procedures and likely effect size within this setting. In addition, given the finding that psychological interventions have greater efficacy with more distressed young people [20], it was considered important to assess whether young people who experienced higher levels of psychological difficulties would gain greater benefit from the intervention than those who did not.

## Aims

1. To test the feasibility of conducting a randomised controlled trial evaluating humanistic counselling in a UK secondary school, identifying:

• likely recruitment rates;

• likely follow-up rates;

• whether trial procedures (screening, assessment, randomisation and allocation to waiting list) would generate any insurmountable ethical or practical problems.

2. To obtain preliminary indications of the efficacy of such an intervention.

3. To examine potential interaction effects between efficacy of intervention and level of mental distress.

## Methods

All procedures in this study received ethical approval from the University of Strathclyde's University Ethics Committee. Informed consent was obtained from young people, and parents/carers gave assent before any screening, assessment and/or intervention procedures were carried out.

### Study design

This was a multi-site, individually randomised controlled study, with participants randomised to either humanistic counselling or to waiting list.

### Participants

Young people were recruited from five secondary schools in total: three in Scotland and two in England, between January and July 2009 (one of these schools in Scotland was unable to continue recruiting from March 2009 due to limited resources, and a third Scottish school was therefore recruited into the project). UK Secondary schools have an intake of pupils aged 11 onwards into the first year of the schooling. The legal minimum leaving age of secondary and post primary schools is 16 years. All schools participating in the research had a pre-existing counselling service, such that the trial-based counselling service acted as an additional provision. The study aimed to recruit 32 participants across the two arms, as recommended for a pilot study of this type by Torgerson and Torgerson [21]. The study focussed on feasibility and no formal power analysis was undertaken. Inclusion and exclusion criteria for participants are given in Appendix 1.

Demographic details for the 27 participants who completed the trial, as taken at baseline assessment, are given in Table 1. In addition to these details, 26 participants described their ethnic origin as white or British (96.3%), with one participant in the waiting list condition indicating a 'mixed background.' One participant in the waiting list group also gave details of a disability, with all other participants considering themselves non-disabled.

**Table 1:** Participant demographics

Client characteristics	Total *N *= 27 (100%)	Counselling *N *= 13 (48%)	Waiting list *N *= (52%)
**Age (years), mean ± SD**	14.20 ± 0.51	14.15 ± 0.56	14.29 ± 0.47

**Gender, n (%)**			

Female	21 (77.8%)	10 (76.9%)	11 (78.6%)
Male	6 (22.2%)	3 (23.1%)	3 (21.4%)

**Meeting MDE cutpoint^bc^**	10 (40%)	5 (41.7%)	5 (38.5%)

**Duration of problems^de^**			

Less than a month	1 (4.5%)	1 (10%)	0 (0%)
1 - 5 months	4 (18.2%)	1 (10%)	3 (25%)

6 - 12 months	4 (18.2%)	3 (30%)	1 (8.3%)

Over a year	13 (59.1%)	5 (50%)	8 (66.7%)

^a^As indicated by SDQ emotion symptoms score at assessment

^b^As indicated by a score ≥ 29 on MFQ at assessment

^c^MFQ data were not completed by two participants

^d^As indicated on the SDQ impact supplement at baseline assessment

^e^SDQ impact supplement data was not completed by four participants

### Interventions

#### Counselling

Young people were offered weekly humanistic counselling for up to six sessions. The nature of the counselling was therapeutic rather than advice- or career-orientated, and was based on the competences for humanistic psychological therapies developed at University College London through funding from Skills for Health [22]. The basic assumption underlying this approach is that people experience emotional and psychological distress when they are estranged from their authentic feelings, needs and preferences [see, [23-26]]. Hence, the principal focus of the humanistic counsellor is on relating to their clients in deeply valuing and understanding ways, such that their clients can come to value and understand themselves and their own experiences more, and find ways of being that are more aligned with their genuine needs and wants. Given these aims, humanistic counsellors tend to work in non-directive ways, listening intently to clients and using the depth of the encounter to understand how they experience their world. Core interventions include reflecting this understanding back to clients; inviting them to access and express underlying emotions and needs; and helping them to reflect on and make sense of their experiences, behaviours and relationships [25].

Counsellors were given copies of the University College London humanistic competences as a manual for practice, and asked to deliver their counselling accordingly. All counsellors were experienced humanistic practitioners who had completed professional, diploma-level trainings in humanistic counselling of approximately 450 hours in duration (generally as part-time study over two years). On average, counsellors had approximately nine years of experience in delivering humanistic therapy, and all counsellors had experience of working with young people in schools. Five counsellors, in total, participated in the trial (one per school). All counsellors were female.

A selection of session recordings was checked by the research team to monitor adherence to humanistic psychological therapy competences. The Humanistic Competences Compliance Checklist Version 3 was developed for this purpose, based on the format of the NICE(R) Record Sheet [27]. Due to the pilot nature of the study, no formal procedure for rating adherence and assessing inter-rater reliability was used. However, all recordings were considered, by the research team, to be compliant with humanistic competences.

Counselling took place during school periods, generally on a weekly basis, with sessions lasting for approximately 45 minutes.

#### Waiting list

Young people allocated to the control condition were not offered any formal counselling intervention. However, they were informed that they had access to the school's full pastoral care provision at any point during the trial, including the school's pre-existing counselling service. At endpoint assessment, participants in the waiting list condition were offered the option of direct entry to counselling.

#### Randomisation

Young people who were eligible to participate in the study following assessment were individually randomised to either counselling for six weeks (intervention) or waiting list (control). To ensure effective concealment, the randomisation sequence was generated by an independent trials unit in blocks of four, stratified by school. Initially, the team had also intended to block randomisation by level of depression (depressed versus non-depressed). However, because of the small numbers of young people entering the trial, and because of uncertainty over how many volunteers would meet criteria for depression, it was decided simply to assess the effect of this variable at analysis. Allocation of participants was accessed by the research team via a dedicated website. Researchers who collected six week endpoint data were blind to the young person's allocation.

#### Measures

The Self-Report Strengths and Difficulties Questionnaire (SDQ) is a widely-used and well-validated [28] brief behavioural screening instrument for children and young people (aged 11 to 16), that can also be used to evaluate the efficacy of specific interventions. Young people are asked to rate 25 items according to how they had been feeling over the past six months (at assessment) and past month (at follow-up), as well as to complete an 'Impact Supplement' assessing overall distress and impairment in different life domains.

The emotional symptoms subscale of the SDQ (SDQ-ES) measures emotional distress, with five items (scored from 0 to 2) assessing levels of physical symptoms, worry, unhappiness, nervousness and fears. It was used as the primary outcome measure for this study as it has been found to be the most responsive of the SDQ subscales to counselling [14]. An SDQ-ES score of 7 to 10 can be interpreted as indicating abnormal levels of emotional symptoms, with a score of 6 indicating borderline levels [29]. Inter-item reliability on the SDQ-ES for the present sample was low to modest (Cronbach's α = .59).

The total difficulties score of the SDQ (SDQ-TS) is generated by summing all the scores on each of the four distress-related scales (emotional symptoms, conduct problems, hyperactivity and peer problems). Inter-item reliability on the SDQ-ES for the present sample was acceptable (Cronbach's α = .76).

The prosocial subscale of the SDQ (SDQ-PS) consists of the remaining five SDQ items, and assesses the young person's perception of themselves as kind and helpful to others. Inter-item reliability on the SDQ-PS for the present sample was modest (Cronbach's α = .62).

The impact score of the SDQ (SDQ-IMP) is derived from a series of items on the Impact Supplement. Inter-item reliability on the SDQ-ES for the present sample was modest (Cronbach's α = .66).

Self-reported change on the SDQ (SDQ-SR) is indicated by one item on the follow-up Impact Supplement which asks the young person to rate their problems 'since coming to the clinic' on a 5-point scale (1 = *Much Worse*, 5 = *Much Better*).

The Young Person's CORE is a 10-item measure of emotional wellbeing for 11 to 16 year olds that has been shown to have acceptable psychometric properties and is sensitive to change [30]. Earlier versions of the YP-CORE measure have been used widely in the evaluation of school-based counselling [14]. Inter-item reliability on the YP-CORE for the present sample was acceptable (Cronbach's α = .80).

The child-report version of the Mood and Feelings Questionnaire (MFQ-C) is a 33 item, well-validated questionnaire designed to detect major depressive episodes (MDE) in children and adolescents [31]. A score of 29 or above has been found to optimally discriminate youth with MDE from those who do not meet criteria for this diagnosis [32] (participants meeting, and not meeting, this cutpoint are subsequently referred to as 'meeting MDE cutpoint' and 'not meeting MDE cutpoint' respectively). Inter-item reliability on the MFQ for the present sample was acceptable (Cronbach's α = .90).

The 'Social Inclusion Questionnaire' (SIQ) is a self-report measure developed by Bury NHS Trust and proposed for use as part of the Improving Access to Psychological Therapies (IAPT) minimum dataset for children and young people. It assesses children's school-related behaviours (such as self-reported school absences in the last month) and attitudes (e.g., 'I am not interested in school'). Items on the SIQ, excepting number of school absences, showed acceptable levels of inter-item (Cronbach's α = .71) and were combined into single 'school wellbeing' variables, with higher scores indicating a more positive attitude towards school and schoolwork.

The Experience of Service Questionnaire (ESQ) is a self-report measure developed by Bury NHS Trust and proposed for use as part of the IAPT minimum dataset for children and young people. It principally consists of 12 items which ask the young person to rate how positively or negatively they experienced the service (for instance, 'I feel the people here know how to help me.'). Items are rated from 0 (*Not true*) to 2 (*Certainly true*), giving a maximum possible score of 24. Items on the ESQ demonstrated acceptable inter-item reliability (Cronbach's α = .88) and were combined into a single 'satisfaction with counselling' variable.

The Attitudes to Counselling questionnaire (ACQ) is a short, purpose-built questionnaire designed to assess young people's interest in participating in the present trial, and to assess their motivation for counselling. The ACQ asks participants, on a four point scale, to indicate: Whether there are things in their life that make them feel sad or worried; Whether they think it would be helpful to talk to someone about this; Whether they would be willing to talk about this to an adult who is professionally trained to help them; and Whether they would be willing to participate in this study? Items on the ACQ demonstrated acceptable levels of inter-item reliability at assessment (Cronbach's α = .72), as well as acceptable levels of test-retest reliability from screening to assessment (ρ = .71). Baseline scores on all four items at assessment were therefore combined into a single 'motivation for counselling' variable.

The Adapted Change Interview is a revision of the Change Interview [33] for use with children and young people. It was developed by a doctoral student, in association with its originator. The Adapted Change Interview asks clients to respond to a series of questions regarding their experience of the counselling intervention, what effect they felt it had, and why they felt it might have impacted upon them (an in depth analysis of these responses are to be published separately, see Lynass, Pykhtina, Cooper: A thematic analysis of young people's experience of counselling in five secondary schools across the UK, submitted). Participants in the waiting list condition were asked to participate in a modified version of this interview protocol, in which they were asked about any change during their waiting for counselling, and factors that may have contributed.

A semi-structured debriefing interview schedule was devised for personnel involved in the trial to assess perceived feasibility.

### Procedures

Researchers attended pupils' Personal, Social and Health Education (PSHE) classes or another equivalent time period (as negotiated with the schools' pastoral care staff), and invited the young people to participate in a brief screening procedure. The screening procedure consisted of the completion of the SDQ and the ACQ.

If a young person indicated on their ACQ that they were willing to participate in the study, the researcher then discussed with a member of the school's pastoral care team the eligibility of that young person (young people who volunteered for the trial were informed that this consultation would take place). If the pastoral care teacher assessed the young person as being capable of giving informed consent for participation in the trial, and if they were viewed as meeting all other relevant criteria (see Appendix 1), the young person was invited to attend an assessment meeting with a researcher. At this meeting, the young person was given further details of the study, and invited to take part in the assessment, randomisation and intervention phases of the study. If they consented to do so, baseline measures were taken, and if the young person continued to meet all criteria, they were accepted into the study and randomised to either counselling or waiting list.

Endpoint measures were taken at approximately six weeks after baseline assessment. Given the difficulties of assessing pupils and delivering interventions outside of school term times, this six week period was defined as six *school *weeks from baseline (whether consecutive or non-consecutive weeks, though not including the summer holidays), rather than six calendar weeks.

To assess the feasibility of trial procedures, debriefing interviews were offered to all personnel involved in the trial: pastoral care staff, researchers, counsellors, and counselling service managers.

### Data analysis

All statistical analyses were conducted using SPSS 17.0.

Descriptive statistical methods were used to identify likely recruitment and attrition rates; and qualitative analysis of interview data was utilised to identify any major ethical and procedural problems.

Given the pilot nature of the trial, missing outcome data were not imputed, and only participants who completed follow up assessments were included in the analyses. Analysis of covariance (ANCOVA) was used for the primary outcome measure and for all secondary outcome measures where baseline and endpoint data were collected and were normally distributed; with endpoint data acting as the dependent variable, baseline data as the covariate, and allocation (counselling versus waiting list) as a fixed factor. Endpoint data only (SDQ-SR) were analysed using analysis of variance (ANOVA), and non-normally distributed data were analysed using a Mann-Whitney Test at endpoint. The potential moderating role of baseline level of depression (met/did not meet MDE cutpoint on MFQ) was assessed by entering this variable into the ANCOVA as an interaction with treatment allocation. The potential predictive role of other variables (gender, age, number of sessions attended, motivation for counselling) for participants in the counselling condition was analysed through ANCOVA.

Effect sizes and 95% confidence intervals were calculated using the Effect Size Calculator from the Centre for Evaluation and Monitoring, Durham University http://www.cemcentre.org/. Effect sizes are given as Hedges' *g *throughout the paper. Like Cohen's *d*, Hedges' *g *is calculated by dividing the difference between experimental and control group means at endpoint by the pooled standard deviation; however, it uses a slightly different formula to calculate the latter [see [34]], correcting for biases that can occur in smaller sample sizes. To describe the magnitude of effect sizes, we have used standardised criteria from Cohen [35] whereby an effect size (Cohen's *d*) of 0.2 can be considered small, 0.5 medium and 0.8 large. Hedges' *g *can be converted to Cohen's *d *for this purpose.

Given the small numbers of participants involved in this pilot trial, all analyses should be considered indicative only and not appropriate as a basis for clinical decision making.

## Results

### Feasibility

**Figure 1:**
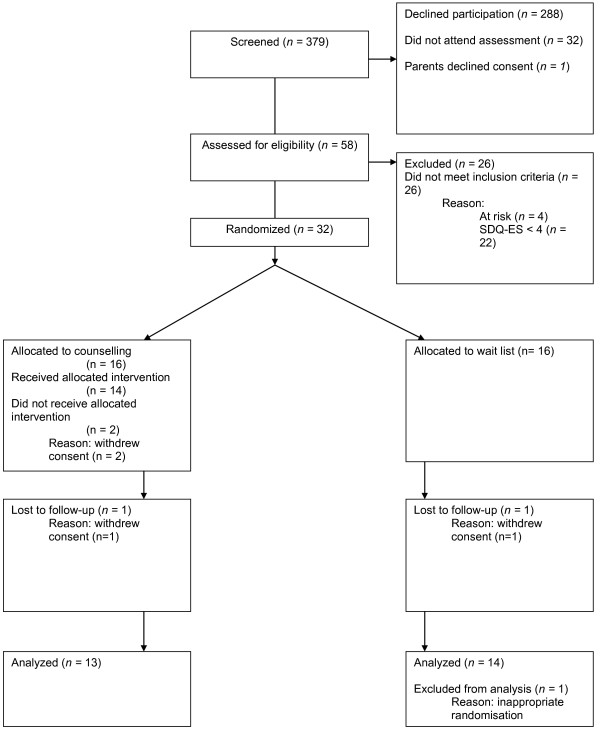
**Participant flow diagram**.

Over two school terms, 379 young people were screened for participation in the trial (see Figure 1). This is approximately 47 young people per school per term (or approximately two classes per school per term), which was acceptable to the schools involved. Fifty-eight of these 379 young people (15.3%) went on to be assessed for eligibility to participate in the study and, of these, 32 (8.2% of those screened) went on to be randomised. This gives a recruitment rate into the trial of 2.7 young people per school per term, or approximately 1.3 young people per class screened. Had the criteria for participation in the trial been set at an SDQ-ES minimum score of 5, 20 young people would have been recruited into the trial, giving a recruitment rate of 5.3% of young people screened, or approximately 0.8 young people per class screened.

Of the 32 young people randomised, 16 were allocated to counselling and 16 to the waiting list condition. In total, four participants declined to participate in endpoint assessment (12.5% withdrawal rate). Three of these were in the counselling condition (18.8% of those allocated to counselling): two pupils withdrew consent shortly after randomisation and one pupil had two sessions before parental assent was withdrawn. The other participant was in the waiting list condition (6.3% of those allocated to waiting list) and withdrew consent shortly after randomisation. None of the participants in the waiting list condition referred themselves, during this six week period, to the school's pre-existing counselling service. In addition, one participant who had been allocated to the waiting list condition was subsequently found to have been wrongly randomised as they failed to meet the defined inclusion criteria (SDQ-ES < 4). After discussion with the trial Steering Group (who were blinded to allocation and outcome), this participant was excluded from any further analysis. Hence, analyses were conducted on 27 participants in total, 13 of whom had been allocated to the counselling condition (48.1% of those randomised), and 14 to the waiting list condition (52%).

Of the 13 participants in the analysis allocated to the counselling arm of the trial, ten attended four sessions of counselling or more (76.9%), and five attended for all six sessions (38.5%). The mean number of sessions attended per participant was 4.54 (*SD *= 1.67).

Ten participants, in total, met or exceeded the MFQ cutpoint for MDE (31.3% of those randomised, Table 1): five in each condition.

Participation in the trial was generally described as positive by clients and by those participating in debriefing interviews, with no major ethical obstacles encountered. However, two practical problems were encountered. First, large numbers of young people were assessed who then needed to be excluded from the trial because of low SDQ-ES scores (*n *= 22, 37.9% of those assessed). Second, three of the young people in the counselling arm of the trial (23.1%) indicated in the Adapted Change Interview that they would have liked more counselling.

### Preliminary indications of efficacy

Table 2 presents changes on the eight primary and secondary outcome measures from baseline to endpoint, and self-reported ratings of change at endpoint. Participants who attended counselling did not improve significantly more on the primary outcome measure, the SDQ-ES, than those on the waiting list (*g *= 0.03).

**Table 2:** Change in baseline to endpoint in psychological distress (*n *= 27)

	Counselling		Waiting list					
**Measure**	**Baseline**	**Endpoint**	**Baseline**	**Endpoint**	**F**	p*	**Effect size (*g*)**	**ES confidence interval (95%)**

SDQ-ES	5.31 (1.55)	4.08 (1.98)	5.43 (1.56)	4.14 (2.21)	0.00	.99	0.03	-0.72 - 0.78

SDQ-TD	16.08 (6.45)	12.46 (5.53)	16.07 (6.44)	13.86 (5.41)	0.84	.37	0.25	-0.51 - 1.01

SDQ-PS	8.00 (1.73)	9.15 (0.69)	8.21 (1.42)	7.86 (1.83)	12.77	.002	0.89	0.10 - 1.68

SDQ-IMP^a^	2.00 (1.94)	1.89 (2.93)	1.36 (1.29)	1.36 (1.50)	0.17	.87	-0.23	-1.11 - 0.65

YP-CORE	17.31 (6.14)	10.46 (7.45)	16.63 (8.20)	12.29 (6.17)	1.1	.30	0.26	-0.50 - 1.02

MFQ	24.67 (12.62)	15.85 (9.34)	22.54 (12.06)	16.06 (10.54)	0.01	.94	0.02	-0.73 - 0.77

SIQ-ABS	1.54 (2.18)	1.54 (1.51)	1.43 (1.57)	2.14 (3.72)		.79	0.19	-0.57 - 0.95

SIQ-SWB	7.85 (2.70)	8.54 (2.67)	8.50 (2.44)	8.64 (2.31)	0.19	.67	-0.04	-0.79 - 0.72

SDQ-SR^b^		3.18 (0.75)		2.45 (1.04)	3.56	.074	0.78	-0.13 - 1.69

Values represent mean (SD). SDQ-ES = SDQ Emotional symptoms scale; SDQ-TD = SDQ Total Difficulties score; SDQ-PS = SDQ Prosocial score; SDQ-IMP = SDQ Impact score; YP-CORE = Young person's CORE; MFQ = Moods and Feelings Questionnaire; SIQ-ABS = school absences; SIQ-SWB = "School wellbeing"; SDQ-SR = SDQ self-rating of improvement (endpoint only)

*ANCOVA was used for all measures in which baseline and endpoint data are available and normally distributed; ANOVA for SDQ-SR; Mann-Whitney Test on endpoint data for SIQ-ABS due to non-normal distribution

^a^SDQ-IMP data were not completed by four participants in the counselling condition and three participants in the waiting list condition

^b^SDQ-SR data were not completed by two participants in the counselling condition and three participants in the waiting list condition

On the secondary outcome measures, clients allocated to counselling showed significantly more improvement than those allocated to waiting list conditions on the prosocial subscale of the SDQ (*g *= 0.89) but not on any of the other measures. At endpoint, there was a trend towards counselling participants rating their improvements over the six week period as greater than those allocated to waiting list conditions (*g *= 0.78).

The mean effect size across the nine outcome measures was 0.24.

For participants in the counselling condition, improvements on the SDQ-ES were not significantly related to gender, age, number of sessions attended, or level of motivation.

All five of the participants in the counselling condition who had met the MFQ cutpoint for MDE moved below the cutpoint at endpoint (100%), as did four of the participants in the waiting list condition (80%). However, in both conditions, one participant who had not met the cutpoint for MDE at baseline assessment moved into the MDE range at endpoint.

The Experience of Service Questionnaire was completed by 11 of the 13 participants in the counselling condition. This indicated high overall levels of satisfaction with the counselling received, with a mean score of 21.91 (*SD *= 3.18). The items most strongly endorsed were 'I felt that the people who saw me listened to me' (*M *= 2) and 'I was treated well by the people who saw me' (*M *= 2). The item least strongly endorsed was 'My appointments are usually at a convenient time' (*M *= 1.45).

### Level of mental distress

**Figure 2:**
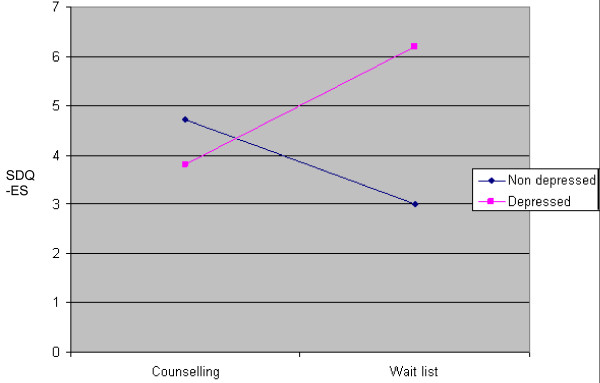
**Predicted post-treatment scores for MDE and non-MDE sample**.

A significant interaction was found between level of distress and treatment allocation (F = 9.69, *p *= .005) (Figure 2). Participants meeting the cutpoint for MDE showed greater change in the counselling condition compared with the waiting list condition, while the reverse was true for participants who were below this cutpoint. Analysis of data from the subgroup of clients who met the MDE cutpoint only (*n *= 10) found a trend towards significantly greater efficacy for counselling over waiting list (*p *= .087), with an effect size (*g*) for treatment against control of 1.13 (95% CI = -0.21 - 2.46) on the SDQ-ES. However, with the very small numbers in this clinical group, this figure must be treated with caution.

## Discussion

The recruitment procedures developed for this pilot study appear to be a viable and robust means of inducting young people into a trial of UK secondary school-based counselling. In addition, attrition rates for randomised participants were acceptable; no major ethical or procedural obstacles emerged; and participants and professionals involved in the trial generally described their experience as rewarding. This suggests that the present protocol could be scaled-up to a fully-powered randomised controlled trial of counselling in schools. However, to reduce the numbers of participants excluded at assessment due to low mental distress scores, it would be advisable to assess only those who demonstrated relatively high levels of emotional distress at screening (for instance, an SDQ-ES score of 4 or more).

With respect to preliminary indications of the efficacy of school-based humanistic counselling, findings were mixed. On the one hand, change on the primary outcome measure indicated that the counselling was not efficacious in reducing levels of emotional distress; and average changes across all outcome measures indicated only a small effect. However, the intervention did bring about significant improvements in prosocial behaviour and there was a trend towards greater self-reported improvements.

One possible explanation for these findings is that humanistic counselling, in general, has a negligible overall effect, as some previous meta-analyses have suggested [8]. However, the significant interaction between amount of improvement and level of distress suggests that this relatively small effect size may be related to the inclusion of participants in the trial with only moderate levels of initial distress. It is a well-established finding in the field of both child and adolescent [20] and adult [36,37] mental health that more distressed clients demonstrate more change. Hence, although the sample size is very small, the present finding of a large overall effect size with young people meeting the cutpoint for MDE suggests that humanistic counselling may prove to have acceptable levels of efficacy if tested within a more severely distressed population. This suggests that, for future studies, it may be advisable to use a more stringent inclusion criterion for levels of mental distress, such as a score of 5 or greater on the SDQ-ES, or SDQ Total Difficulties within the abnormal range.

Given, however, that many of the young people who enter school-based counselling do not have such high levels of emotional or psychological distress [14], the present findings may suggest that such an intervention is not appropriate for this population. However, an alternative possibility is that it is helpful, but in ways that are not picked up by standardised measures of emotional and psychological distress. Support for such an interpretation comes from four findings in the present study. First, there was a trend for counselling participants to indicate significantly more improvements than those in the waiting list condition when problems were self-defined (SDQ-SR). Second, those with lower initial levels of psychological distress reported just as much satisfaction with the counselling as those with higher levels. Third, responses to the Adapted Change interviews (Lynass, Pykhtina, Cooper: A thematic analysis of young people's experience of counselling in five secondary schools across the UK, submitted) indicated that the most frequent changes following counselling were to do with greater feelings of wellbeing and improved relationships, rather than direct reductions in levels of psychological distress. Fourth, significant positive improvements in the counselling condition were found on the prosocial subscale of the SDQ. For future trials of humanistic counselling which involve non-clinical populations, then, it may be valuable to include more personalised measures of psychological change [such as the Goal Based Outcome measure, [38]], as well as measures that focus on positive mental wellbeing [39] and interpersonal relating.

Another possible explanation for the overall low effect size for counselling is the brevity of the period between assessment and endpoint. This was set at six weeks as an ethical safeguard for young people allocated to the waiting list condition, who may have found a longer period unacceptable. However, young people participating in the control arm of this trial did not report feeling disadvantaged by this allocation, and did not self-refer to the pre-existing school-based counselling service. In addition, around a quarter of the young people receiving counselling indicated that they did not feel they had completed their work within the six week limit. For these reasons, for future research, we would suggest that it is appropriate to extend the intervention period to a school term (10 to 12 weeks).

Finally, in attempting to understand the relatively low overall efficacy of counselling in the present trial, it is worth noting that participants in the waiting list condition appear to have fared relatively well, and considerably better than control participants in similar trials [e.g., [39-41]]. Evidence from the Adapted Change Interview with waiting list participants suggests two reasons for this. First, they tended to experience the assessment interview as a very helpful intervention in itself. Second, the promise of counselling in a relatively short period of time (six weeks) tended to instil in them a considerable degree of hope, expectation and motivation which, in itself, has been found to be of considerable benefit [40,41]. Although such factors would be of relevance in any psychological therapies trial, the relative brevity of the current intervention may have made them proportionately more significant. Again, this would suggest that the present design would benefit from a longer period between baseline assessment and endpoint.

The low to modest alpha coefficient of the SDQ subscales in the present study, including the primary outcome indicator (SDQ-ES), is something of a concern. This may reflect the limited length of the 5-item SDQ subscales, and has been identified as a problem in other studies of the SDQ's psychometric properties [42,43]. For future studies, therefore, measurement of the primary outcome may benefit from a longer measure to maximise reliability.

With respect to other limitations, the small sample size in this pilot means that all outcome findings must be treated with extreme caution. Confidence intervals are wide for all outcome indicators, and a non-equivalent distribution of participants across the two conditions is quite possible. The lack of formal procedures for rating adherence and assessing inter-rater reliability is also an important limitation, and means that the exact nature of the intervention being delivered cannot be verified. Findings from the Adapted Change Interview should be treated with particular caution given that the unstructured nature of the response format may have led participants to provide more socially desirable responses. A final limitation of the present study is the lack of extended follow-up.

### Recommendations

Although, with respect to efficacy, the present findings are mixed, given the proliferation of school-based humanistic counselling services in the UK, we believe that it is essential to undertake a fully-powered RCT of this intervention. The procedures developed in the present trial are a viable means by which to conduct such a study. However, we would recommend the following modifications:

• Adopt a higher inclusion criterion for level of mental distress;

• Assess only those young people who, at screening, indicate relatively high levels of mental distress;

• Extend the period from baseline to endpoint to a full school term (approximately 10 to 12 weeks);

• Incorporate measures of wellbeing, interpersonal functioning, and a personalised measure of change;

• Use a longer primary outcome measure to ensure inter-item reliability.

## Conclusion

A viable means of evaluating the efficacy of school-based counselling has been established. This protocol, with some modifications to outcome measures, screening procedures, time span, and inclusion criterion, can be extended to a fully-powered trial. Counselling was not found to bring about improvements in emotional symptoms in young people (*g *= 0.03) and, on the basis of these findings, cannot be indicated as an alternative intervention for CBT for depression. However, prosocial behaviour was increased considerably through the intervention and there were some indications of greater efficacy for more distressed young people. Given the planned dissemination of school-based counselling across the UK, and the mixed findings from the present trial, a fully-powered study, based on the present design, is recommended in order to assess whether or not this intervention is effective in improving levels of psychological wellbeing.

## Competing interests

NR is Director of Research, Policy, and Professional Practice at the British Association for Counselling and Psychotherapy (BACP). KC is the Senior Lead Advisor at BACP, with specific responsibility for Children and Young People.

## Authors' contributions

Each of the first five authors made significant contributions to the design and implementation of the study, with MC and NR as lead researchers. KR worked at BACP as a Research Facilitator in the early stages of the trial. The drafts of the manuscript were written by MC, with preparation and analysis of the data by KMcA and MC. All authors gave critical comments to the manuscript, and have given it final approval.

## Appendix 1: Inclusion and exclusion criteria

Young people were included in the study only if they met all of the following criteria

• Aged 13 to 18.

• Experiencing, at minimum, moderately high levels of emotional distress, as indicated by a score of 4 or above on the SDQ emotional symptoms subscale at assessment.

• Motivated to attend counselling, as indicated by a response of 'Somewhat True' or 'Certainly True' on the ACQ at assessment.

• Capable of consenting to participate in research, as indicated by a member of the pastoral care team.

• Greater than 85 per cent attendance at school, as indicated by a member of the pastoral care team.

Young people were excluded from the study if they met any of the following criteria

• Risk of significant harm to self or other, as indicated by a member of the pastoral care team and the researcher at assessment.

• Involvement with other child and young people mental health agencies, including the established school counselling service, as indicated by a member of the pastoral care team and/or the young person at assessment.

• Planning/likely to move school during period of study, as indicated by a member of the pastoral care team and/or the young person at assessment.
